# Chemiluminescent Optical Fiber Immunosensor Combining Surface Modification and Signal Amplification for Ultrasensitive Determination of Hepatitis B Antigen

**DOI:** 10.3390/s20174912

**Published:** 2020-08-31

**Authors:** Xuexue Xu, Rongbin Nie, Jingwen Huang, Li Yang

**Affiliations:** Key Laboratory of Nanobiosensing and Nanobioanalysis at Universities of Jilin Province, Department of Chemistry, Northeast Normal University, 5268 Renmin Street, Changchun 130024, China; xuxx493@nenu.edu.cn (X.X.); nierb788@nenu.edu.cn (R.N.); huangjw561@nenu.edu.cn (J.H.)

**Keywords:** optical fiber immunosensor, surface modification, signal amplification, hepatitis B surface antigen, hepatitis B e antigen

## Abstract

Optical fiber based immunosensors are very attractive for biomarker detection. In order to improve the sensor response, we propose a promising strategy which combines porous-layer modification of the fiber surface and streptavidin-biotin-peroxidase nano-complex signal amplification in chemiluminescent detection. Two hepatitis B antigens, hepatitis B surface antigen (HBsAg) and hepatitis B e antigen (HBeAg), are used as the targets for analysis using the proposed sensor. Comparing to immunoassays using normal optical fiber sensors, the response of the present sensor is enhanced by a factor of 4.8 and 6.7 for detection of HBsAg and HBeAg, respectively. The limit-of-quantitation of the proposed method is as low as 0.3 fg/mL (0.01 fg/mL) with a wide linear response range of 3 fg/mL–150 ng/mL (0.1 fg/mL–160 ng/mL) for sensing HBsAg (HBeAg). Quantitative determination of HBsAg and HBeAg in human serum samples is performed, showing the applicability of the proposed method for biomarker detection.

## 1. Introduction

Being the causative agent of hepatitis B, early diagnosis of Hepatitis B virus (HBV) infection is very important for treatment of the disease. Hepatitis B surface antigen (HBsAg) and Hepatitis B e antigen (HBeAg) are two reliable indicators and biomarkers for the diagnosis of HBV infection, which would appear in the serum with high infectivity in the early stage of the disease [1,2,3,4,5,6,7]. Accurate detection of these biomarkers in serum samples would be of great value in clinical applications. While enzyme-linked immunosorbent assay (ELISA) is generally regarded as an effective method, developing more convenient sensors is still highly required in order to achieve rapid, sensitive and selective determination of the hepatitis B antigens [8,9,10,11,12,13,14,15,16,17].

Optical fiber immunosensors have attracted intense research interest recently because of their advantages of high sensitivity, simple structure and operation, little susceptible to electromagnetic interference, and easy miniaturization. These sensors use optical fibers as both sensing and light-transmission media, thus are particularly suitable for sensitive optical detection methodologies, for example, chemiluminescent or laser-induced fluorescence (LIF) detection [18,19]. One inherent limitation is that an optical fiber has one-dimensional cylindrical geometry, leading to limited effective surface area. Thus, the load of antibodies on the sensor and the detection sensitivity would be affected. In order to expand the application of optical fiber immunosensors in the detection of low-level biomarkers, many studies have been devoted in recent years to improve the response of the sensors by increasing the surface area, mainly through strategies of surface modification or using porous optical fibers [20,21,22,23,24,25,26,27,28,29,30,31,32,33,34].

Here we propose a promising optical-fiber sensor for ultrasensitive detection of hepatitis B antigens. We introduce porous-structured layer to the fiber surface as the support for immobilization of antibodies, which would enhance the effective surface area, and a biotin-streptavidin nano-complex in the immunoassays which could amplify the chemiluminescent signal. Similar approach has also been employed in our recent study to develop micro-capillary based sensors [35]. With the combination of the dual strategies, the detection sensitively of the optical-fiber sensor is improved significantly. Both HBsAg and HBeAg in human serum samples are accurately determined using the proposed method. The results have been compared with those from traditional ELISA method, which show the proposed immunoassays are promising for detection of hepatitis B antigens in real samples.

## 2. Materials and Methods

### 2.1. Reagents and Materials

Optical fibers were purchased from Wyoptics Technology Co., Ltd. (Shanghai, China) with a core-diameter of 400 μm. The fiber core is surrounded by a 20 μm silica cladding and a 130 μm-thick acrylic-jacket layer. The refractive index of the core and the silica cladding at 633 nm was 1.457 and 1.439, respectively. Standard antigens HBsAg (1.75 mg/mL in antigen diluent) and HBeAg (2.8 mg/mL in antigen diluent), capture antibodies for the antigens (Ab_1_, 2.4 mg/mL for HBsAg and 3.5 mg/mL for HBeAg in antibody diluent), biotinylated antibodies (biotin-Ab_2_, 5 mg/mL in antibody diluent), streptavidin (5 mg/mL in phosphate buffered saline) and biotinylated horseradish peroxidase (HRP) were provided by Lingchao Biological Technology Co., Ltd., (Shanghai, China). HexadecyItrimethylammonium bromide (CTAB), urea, and tetraethyl orthosilicate (TEOS) were supported by Aladdin Chemical Co., Ltd. (Shanghai, China). Other chemicals, including 3-aminopropyl diethoxymethylsilane (3-ADMS), oxidized glutathione (GSSG), glutaraldehyde (GA, 50% aqueous solution), cyclohexane, n-pentanol, bovine serum albumin (BSA), interleukin 6 (IL-6), and cardiac troponin I (cTnI), were purchased from Sigma Chemical (St. Louis, MO, USA). All reagents were of analytical grade and were used without further purification. A chemiluminescent kit (SW2010 Western Blotting ECL) provided by Beijing Solarbio Science and Technology Co. (Beijing, China) was used to record the chemiluminescent signal of the immunoassays. Phosphate buffered saline (PBS, 0.01 M, pH 7.4) was used as the washing buffer which was sterilized and prepared freshly.

### 2.2. Fabrication of Porous-Layer Modified Optical Fiber Immunosensor

To introduce a porous-structured layer on the fiber surface, we need to remove first the acrylic coating and the silicon dioxide coating of the fiber. First, one end of the fiber (about 2 cm) was immersed into chloroform for 10 min. After carefully removing the acrylic protective layer using a knife, the fiber end was soaked in hydrofluoric acid (24%) for 20 min to remove the silicon dioxide cladding via chemical corrosion. Then it was washed successively with 0.1 M NaOH for 1 h and water for 15 min. After drying under N_2_, a porous layer was fabricated on the fiber surface through an in situ two-phase reaction, the procedure of which was similar as that reported in our previous study [36]. Briefly, the pre-treated fiber was inserted in the reaction solution containing 0.05 g CTAB, 0.03 g urea, 1.5 mL H_2_O, 46 μL n-amyl alcohol, 1.5 mL cyclohexane, and 50 μL TEOS. By condensed and refluxed with a stirring speed of 450 RPM for 16 h, the porous layer would grow on the fiber surface. After rinsing with H_2_O and ethanol for 10 min followed by drying with N_2_ for 1 h, the fiber was ready to be used to prepare the optical fiber immunosensor.

In order to introduce amino-group on the surface of the porous-layer optical fiber, the fiber end was first immersed in 3-ADMS toluene solution (1% (*v*/*v*) for 1 h, washed by toluene for 15 min, and dried by N_2_ for 1 h. Then the fiber was immersed in GA solution (2.5% (*v*/*v*)) for 2 h and washed with water for 20 min. Subsequently, the fiber was immersed in 40 μg/mL Ab_1_ and placed in a refrigerator at 4 °C overnight, followed by rinsing with PBS. The unbound aldehyde groups on the fiber surface could link to the amino group of Ab_1_. Finally, the optical fiber was immersed in GSSG (10 mg/mL) for 2 h, then washed with PBS, and stored in a 4 °C when not in use.

### 2.3. Immunoassays Using the Porous-Layer Optical Fiber Sensors with Signal Amplification

**Scheme 1:**
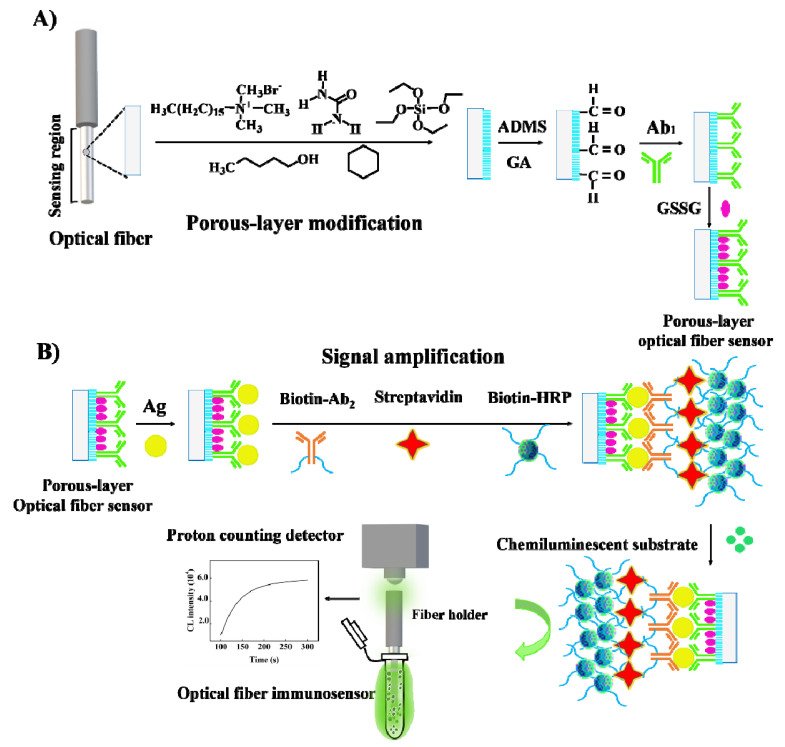
(**A**) The procedure of the fabrication of porous-layer modified optical fiber sensor. (**B**) The procedure of immunoassays with signal amplification.

The procedure of immunoassays using the proposed porous-layer optical fiber sensors is schematically presented in Scheme 1, including sandwich immunoreaction and chemiluminescent detection. Streptavidin-biotin-HRP nanocomposite is employed for chemiluminescent signal amplification [37]. The procedure contains four specific steps: (i) The antigen (Ag) specifically binds to the immobilized antibody (Ab_1_) on the porous surface of the optical fiber; (ii) The biotinylated antibody (biotin-Ab_2_) is immobilized on the fiber via specific Ag-Ab recognition; (iii) The nanocomposite (streptavidin-biotin-HRP) is formed by combining streptavidin with HRP-labeled biotin and then binding to biotin-Ab_2_ on the fiber surface. This composite structure is conducive to signal amplification; (iv) Finally, chemiluminescent signal is detected to record the sensor response. The detailed experimental conditions are described below.

Firstly, the optical fiber sensor was immersed with hepatitis B antigen standard (HBsAg or HBeAg) or clinical sample, incubated at 37 °C for 1 h, and then washed with PBST to remove non-specific physical adsorption. Then the fiber was inserted in 10 μg/mL Biotin-Ab_2_ solution with incubation at 37 °C for another 1 h, allowing the sandwich immunoreaction. After being rinsed with PBST, the fiber was immersed in a streptavidin-biotin-HRP nanocomposite solution (1.25 μg/mL streptavidin and 2.5 μg/mL HRP-biotin). After incubating at 37 °C for 30 min, the optical fiber was rinsed with PBST. Finally, the sensing region of the fiber was immersed in a 1:1 mixed solution of luminol and hydrogen peroxide, and a photon counting detector (CH326 module, Beijing Hamamatsu Photonic Technology Co., Ltd., Beijing, China) was used to measure the chemiluminescent signal. The sensor response is defined as difference of the chemiluminescent intensity of the target sample and that of the blank solution. The total time of the immunoassay for one sample was less than three hours.

### 2.4. Specificity and Recovery Measurements

In order to evaluate the specificity of the porous-layer optical fiber immunosensor, we added an interfering substance (5 μg/mL) to the HBsAg or HBeAg standard solution (50 ng/mL) at a ratio of 1:100. The interferences investigated in the study included bovine serum albumin, alpha-fetoprotein, interleukin-6, carcinoembryonic antigen, procalcitonin, and cardiac troponin. The standard HBsAg or HBeAg solution without any interference was used as a control sample. For the measurement of recovery, standard HBsAg or HBeAg solutions were spiked into normal human serum samples. The concentrations of HBsAg were 0.25, 0.5, 1.0, 5.0, or 10.0 ng/mL, and the concentrations of HBeAg were 0.2, 0.5, 1.0, 5.0, or 8.0 ng/mL.

### 2.5. Analysis of Human Serum Samples

The porous-layer optical fiber immunosensor was applied for determination of HBsAg or HBeAg in clinical human serum samples, which were provided by a hospital health examination center (Changchun, China). Each sample was measured three times. The obtained results were compared with those obtained by the hospital clinical laboratory using the Roche-ECL method (traditional ELISA method) to obtain a correlation curve.

## 3. Results and Discussion

### 3.1. Characterization of the Porous-Layer Optical Fiber

**Figure 1:**
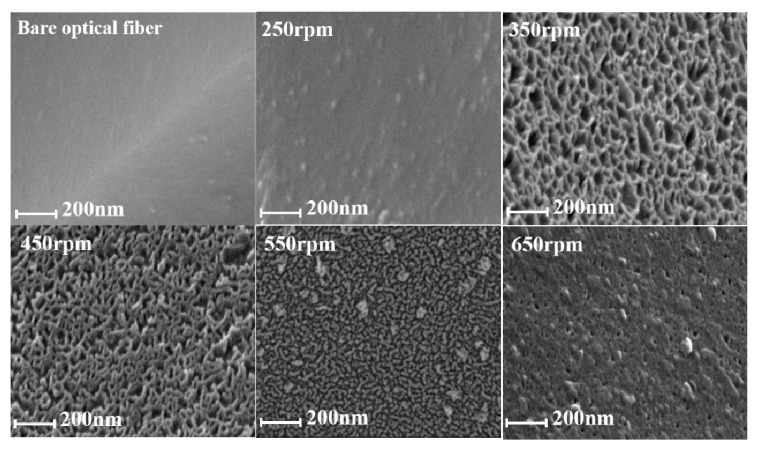
Scanning electron microscope (SEM) images of the surface of the bare optical fiber and porous-layer modified fiber with different magnetic stirring speed.

In order to improve the response of the optical fiber immunosensor, we modified the fiber surface with a uniform porous layer, so as to enhance the specific surface area of the optical fiber which serves as a carrier for immobilizing antibodies. We investigated the growth of the porous layer on the fiber surface at different magnetic stirring speeds in the range of 250 rpm to 650 rpm, and characterized the surface morphology with scanning electron microscope (SEM) images. Figure 1 shows the SEM images of a bare optical fiber and the porous-layer modified optical fibers obtained at different stirring speeds. The surface of the bare optical fiber is quite smooth, but after two-phase reaction at different speeds, the surface coverage is different. It can be seen from Figure 1 that the thickness of the porous layer changes with the increase of stirring speed. At the low stirring rate, the hydrolysis rate of the precursors and the diffusion rates of resultant silicates from oil phase to aqueous phase are relatively low, resulting in small pore size and thin shell. When the stirring rate increases to 450 rpm, oil and water phases remain well separated, while the diffusion and hydrolysis rates of silicates increase significantly, promoting the growth of the fibrous mesostructure into a thicker shell with larger pore size. When the stirring rate is further increased to 650 rpm, the mixture changes from a well separated biphase to a microemulsion phase, in which the diffusion and hydrolysis rates of silicates would be the highest. Therefore, both too fast and too slow mass transfer are not good for the growth of the fibrous structure. A stirring speed of 450 rpm is selected for fabrication of the sensor in the study.

**Figure 2:**
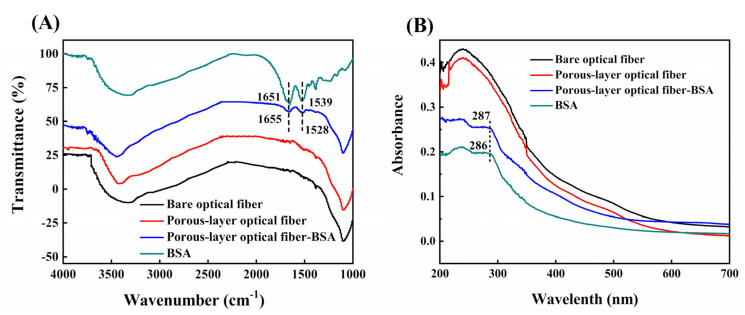
(**A**) FT-IR spectrum and (**B**) UV-vis spectrum of a bare optical fiber (black line), a porous-layer modified optical fiber (red line), a bovine serum albumin (BSA)-coated porous-layer modified optical fiber (blue line) and free BSA (green line).

For characterization the successful immobilization, we should compare the spectroscopic results with those of pure free antibodies. The antibodies used in the present study, however, are commercially purchased with complex matrix which would affect the spectroscopic measurements. Concerning that antibodies are proteins and the mechanism for immobilization is exactly the same, we use bovine serum albumin (BSA) as test protein instead of antibodies and characterize the BSA-immobilized optical fiber by FT-IR and UV-vis spectroscopy. To record FTIR and UV spectrum of BSA-immobilized fibers, a 2 cm-fiber with BSA immobilization was cut into small sections with a fiber cutting knife and then ground into powder in a mortar for spectroscopic measurements. Figure 2A,B shows the UV-vis and FT-IR spectra of free BSA samples, a bare optical fiber and porous-layer modified optical fibers with or without immobilization of BSA. As shown in Figure 2A, as BSA is immobilized on the optical fiber, two peaks at 1655 cm^−1^ and 1528 cm^−1^ appear in the FTIR spectrum, which are characteristic vibrational bands of proteins, i.e., the C=O stretching vibration of the protein primary amide and the N-H bending vibration of the secondary amide, respectively. As shown in Figure 2B, both the free BSA sample and the BSA immobilized optical fibers show a characteristic absorption peak of protein around 286 nm, which is absent in bare and porous-layer optical fibers without BSA immobilization. These spectroscopic results confirm that the proteins are successfully immobilized on the fiber surface using the proposed procedure.

### 3.2. Analytical Performance of Immunoassays Using the Optical Fiber Sensors

**Figure 3:**
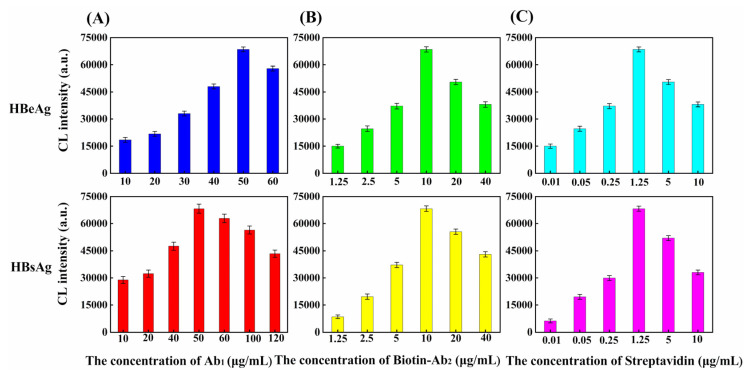
Effect of the immunoassay conditions on the response of the optical fiber-based sensor. (**A**) Ab_1_ concentration (**B**) Biotin-Ab_2_ concentration, and (**C**) streptavidin concentration.

Several key conditions involved in the immunoassays are investigated and optimized, including the concentrations of both Ab_1_ and Ab_2_, and the molar ratio of streptavidin to enzyme-labeled biotin used for signal amplification. In Figure 3A,B, we show the dependence of the chemiluminescent intensity on the concentration of Ab_1_ and Biotin-Ab_2_, respectively. For analysis of either HBeAg or HBsAg, the response of the sensor first increases upon increasing the concentration of either antibody, but turns to decrease if the antibody concentration is too high. This could be attributed to fact that the steric hindrance becomes significant at high antibody concentration, which will affect the ability to capture the target and perform non-specific adsorption. In other words, the binding site of the molecule is fixed, and the space on the carrier is also fixed. With the increase of molecular concentration, the space will become crowded, thus the repulsion force between the molecules will become larger and larger, and the response signal will be inhibited and thus reduced. The same explanation is also applicable to Ab_1_ and Biotin-Ab_2_. We use the optimal concentrations of antibodies for detection of the analytes and obtain the standard curves. The concentration of Ab_1_ is not adjusted during the real sample analysis. For the signal amplification, the molar ratio of streptavidin to HRP-biotin is kept at 1:4 because one streptavidin molecule can bind four biotin molecules. The effect of streptavidin concentration on the sensor response is shown in Figure 3C, with the addition of the signal amplification, the chemiluminescence intensity increases rapidly, and the optimal streptavidin concentration is 1.25 μg/mL, for analysis of both antigens. We also investigate the dependence of the response on these conditions, for bare optical fiber sensor with and without signal amplification, as well as porous layer modified optical fiber sensor without signal amplification. As shown in Figures S1 and S2, a similar tendency of the response of each sensor on the assaying conditions is observed as those in porous layer modified optical fiber sensor with signal amplification.

**Figure 4:**
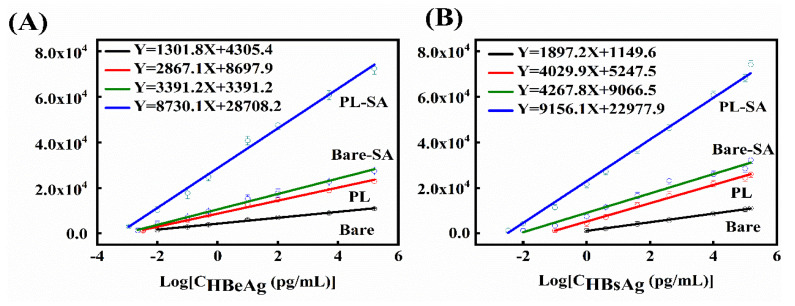
Semilogarithmic curve of the chemiluminescent intensity vs the concentration of Hepatitis B e antigen (HBeAg) (**A**) and hepatitis B surface antigen (HBsAg) (**B**), for a porous-layer modified optical fiber sensor with signal amplification (PL-SA), a PL fiber sensor without SA (PL), a normal optical-fiber sensor without SA (bare) or with SA (bare-SA). Each data point is an averaged result of three replicated assays. Linear fitting of each curve is indicated in the figure.

We evaluated the linear range and limit-of-detection of the proposed immunoassays. The semilogarithmic curve of the chemiluminescent intensity vs the concentration of hepatitis B antigen is determined, as shown in Figure 4A,B for HBsAg and HBeAg, respectively. A wide linear range for detection is achieved with antigen concentration over eight orders of magnitude, and the limit of quantification (LOQ), determined at a signal-to-noise ratio of 10, is as low as 0.01 fg/mL for HBeAg and 0.3 fg/mL for HBsAg. For comparison, we also present in the figure the results for other three immunoassays, i.e., bare optical fiber sensor with/without signal amplification (Bare-SA/ Bare), and porous layer modified optical fiber sensor without signal amplification (PL). The modification steps of Bare-SA immunoassay are the same as those of porous-layer modified optical fiber sensor with signal amplification (PL-SA). except for the step of increasing porosity. It is clearly seen that using either surface modification by porous layer or signal amplification, the linear range and LOQ of optical fiber sensors can be improved. The response of the present approach which combines both strategies, is enhanced by a factor of about 6.7 times (for HBeAg) and 4.8 times (for HBsAg) compared to that of bare optical fiber sensors. Our results show that with the combination of porous layer surface modification and signal amplification of streptavidin-biotin nanocomposite, the developed optical fiber immunosensor has a large detection range and ultra-high sensitivity for detection of HBeAg and HBsAg.

**Figure 5:**
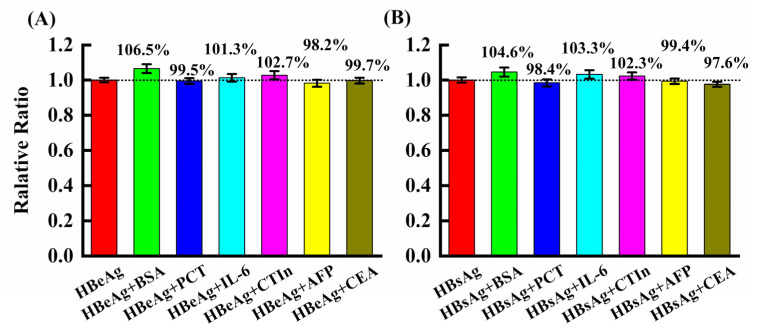
Interference effects from various substances in 50 ng/mL HBeAg (**A**) or HBsAg (**B**) solution. The concentration of each substance is 5.0 μg/mL. Error bars (standard deviation of three replicated assays).

The specificity of the present immunoassays of HBsAg and HBeAg is investigated by using various samples, including alpha-fetoprotein, carcinoembryonic antigen, cardiac troponin, bovine serum albumin, interleukin 6 and procalcitonin as interference. In Figure 5, we show the relative ratio of each sample to the target antigen. The results show the relative ratio of HBeAg and HBsAg in the samples containing the interfering substances is in the range of 98.2–106.5% and 98.4–104.6%, respectively, indicating the method has satisfactory selectivity in detecting the target in complex samples. The different batches of sensors have been used to evaluate the repeatability of the proposed method. The RSD (*n* = 3) is less than 3.2% and 4.1% for intra-day and inter-day analysis respectively, showing good repeatability of the immunoassays in detecting hepatitis B antigens.

### 3.3. Analysis of Serum Samples

Standard samples spiked with different concentrations of the target antigens are analyzed using the proposed method. The results are listed in Table 1 and Table 2 for HBsAg and HBeAg, respectively. The recovery for detecting HBsAg is 95.0–104.2% with RSD (*n* = 3) of 4.3–10.7%, while that for HBsAg is 91.0–106.0% with RSD (*n* = 3) of 4.7–9.6%. The results of the spiking measurements show that the proposed sensor can be used for accurate determination of trace HBsAg and HBeAg in real samples.

**Figure 6:**
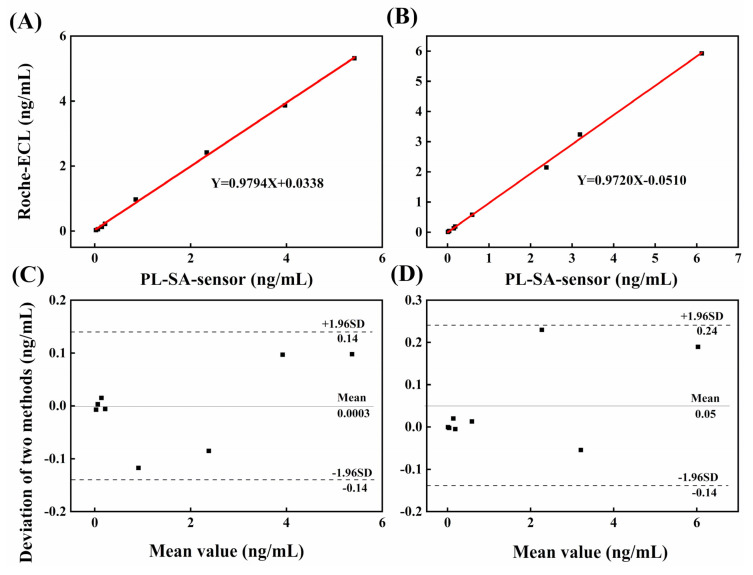
Correlation and Bland-Altman analysis for the agreement between the proposed PL-SA-sensor and the Roche-ECL tests for the immunoassay of HBeAg (**A**,**C**) and HBsAg (**B**,**D**) in human serum samples. Data shows a 95% confidence interval of the mean for 8 serum samples.

Eight clinical human serum samples provided by the hospital are analyzed using the present method, and the results are compared with those of the Roche-ECL method which are independently measured by the hospital laboratory. The results are listed in Tables S1 and S2 in the Supplementary Material. The relationship of our results and the Roche-ECL results is shown in Figure 6. It is found that the two methods have a good correlation, with the correlation coefficients of 0.9794 and 0.9720 for HBsAg and HBeAg. Our results show the capability of the present method for assay of HBsAg and HBeAg in clinical samples.

## 4. Conclusions

In this study, we present a promising method that can significantly improve the response of optical fiber based immunosensors for detection of two hepatitis B antigens, HBsAg and HBeAg. For fabrication of the sensor, we modify the fiber surface with a porous-structured layer, which can enhance the effective surface area thus the loading of antibodies. For immunoassays, we utilize chemiluminescent detection with streptavidin-biotin-peroxidase nano-complex to amplify the signal. With combination of surface modification and signal amplification, the response is enhanced by 4.8 and 6.7 times for detection of HBsAg and HBeAg, respectively. Our method exhibits extremely low LOQ down to 0.01 fg/mL, and a wide linear range with antigen concentration over eight orders of magnitude. The successful application of the proposed immunosensor in quantitative determination of HBsAg and HBeAg in human serum samples has been achieved with satisfactory accuracy. Our study provides a stable and versatile method that can be used for ultra-sensitive detection of various trace targets in complex biologic samples. Future research will focus on the combination of the optical-fiber-based immunosensor and micro-devices to realize high-throughput analysis and potable-of-care testing.

## Figures and Tables

**Table 1:** HBeAg recoveries in serum samples using the PL-SA-sensors.

Spiked Value	Measured Value	Recovery	RSD (*n* = 3)
(ng/mL)	(ng/mL)	(%)	(%)
0	0.22	--	10.7
0.2	0.41	95	7.3
0.5	0.74	104	5.6
1	1.18	96	6.9
5	5.43	104.2	8.4
8	8.11	98.6	4.3

**Table 2:** HBsAg recoveries in serum samples using the PL-SA-sensors.

Spiked Value	Measured Value	Recovery	RSD (*n* = 3)
(ng/mL)	(ng/mL)	(%)	(%)
0	0.14	--	9.6
0.25	0.37	91	5.8
0.5	0.67	106	8.9
1	1.1	96	4.7
5	5.39	105	7.1
10	9.68	95.4	5.5

## Supplementary Materials

The following are available online at https://www.mdpi.com/1424-8220/20/17/4912/s1, Figure S1: the effects of assay conditions on the responses of different sensors for detection of HBeAg, Figure S2: the effects of assay conditions on the responses of different sensors for detection of HBsAg, Table S1: Detection of HBeAg in serum samples using the PL-SA-sensors and Roche-ECL, Table S2: Detection of HBsAg in serum samples using the PL-SA-sensors and Roche-ECL.
