# Supplementary material for: Chemiluminescent Optical Fiber Immunosensor Combining Surface Modification and Signal Amplification for Ultrasensitive Determination of Hepatitis B Antigen
Source: Sensors (Basel). 2020 Aug 31;20(17):4912. doi: 10.3390/s20174912 (PMC7506923; doi:10.3390/s20174912)
Supplement: Supplementary file 1 [file sensors-20-04912-s001.pdf]

Supplementary Material for

Chemiluminescent Optical Fiber Immunosensor Combining Surface Modification and Signal Amplification for Ultrasensitive Determination of Hepatitis B Antigen

Xuexue Xu, Rongbin Nie, Jingwen Huang, Li Yang*

Key Laboratory of Nanobiosensing and Nanobioanalysis at Universities of Jilin Province, Department of Chemistry, Northeast Normal University, 5268 Renmin Street, Changchun, Jilin Province 130024, PR China

* Corresponding authors: L. Yang, yangl330@nenu.edu.cn, Tel: +86-431-85099762, Fax: +86-431-85099762

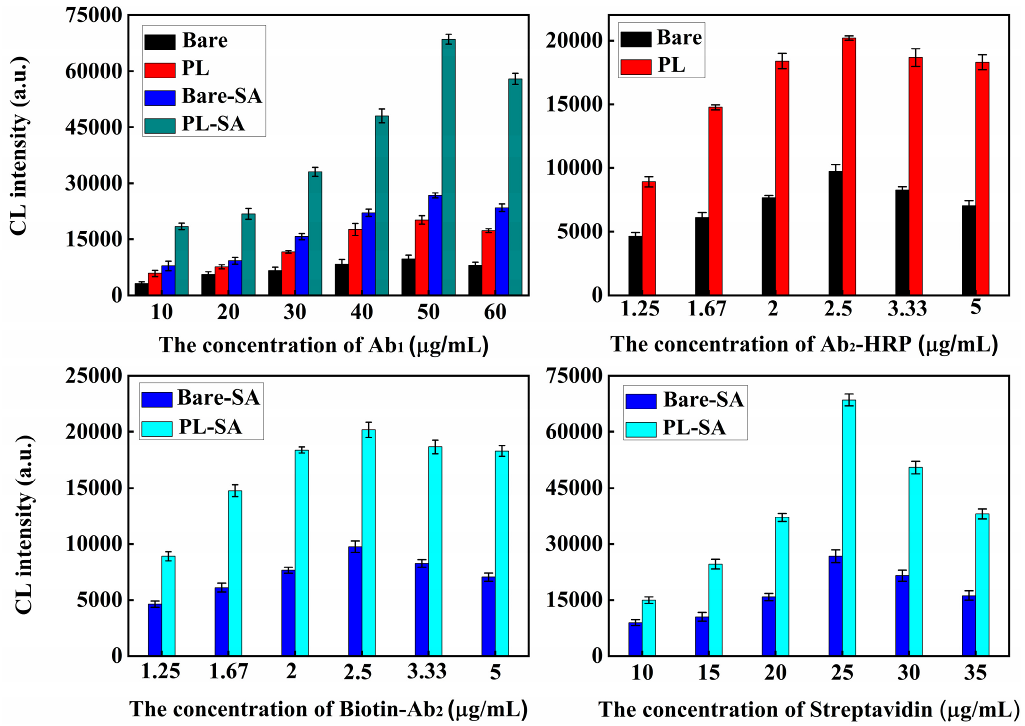

Figure S1. The effects of assay conditions on the responses of different sensors for detection of HBeAg.

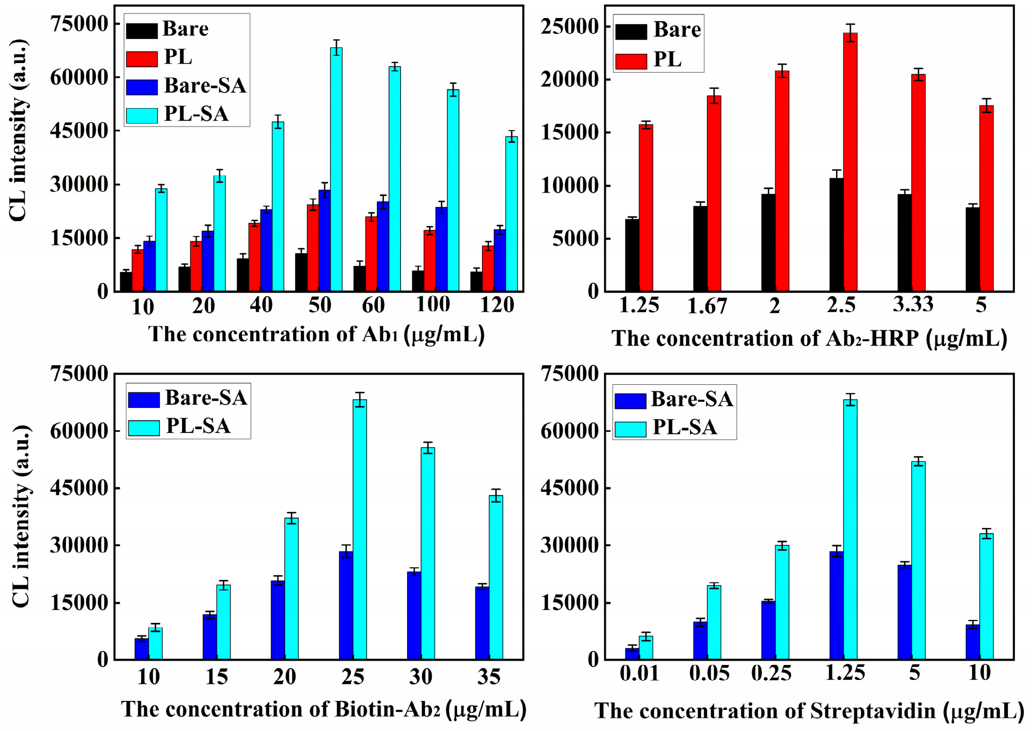

Figure S2. The effects of assay conditions on the responses of different sensors for detection of HBsAg.

Table S1. Detection of HBeAg in serum samples using the PL-SA-sensors and Roche-ECL.

Sample number	Assay results[a] (ng/mL)	RSD (n=3)[a] (%)	Roche-ECL[b] (ng/mL)	Relative error[c] (%)
1	0.023	11.3	0.03	-2.3
2	0.063	8.4	0.06	6.6
3	0.145	7.3	0.13	11.5
4	0.214	12.4	0.22	-2.7
5	0.853	9.8	0.97	-12.1
6	2.335	6.1	2.42	-3.5
7	3.967	2.7	3.87	2.5
8	5.418	5.2	5.32	1.8

[a] assay using PL-SA-sensors.

[b] independently measured by clinical laboratory of hospitals.

[c] the difference of the assay and clinical results divided by the clinical results.

Table S2. Detection of HBsAg in serum samples using the PL-SA-sensors and Roche-ECL.

**Sample** number	**Assay results**[a] (ng/mL)	**RSD (n=3)**[a] (%)	**Roche-ECL**[b] (ng/mL)	**Relative error**[c] (%)
1	0.0098	14.2	0.01	-2.0
2	0.038	10.4	0.04	-5.0
3	0.150	2.8	0.13	15.4
4	0.185	3.9	0.19	-2.6
5	0.593	9.3	0.58	2.2
6	2.380	7.9	2.15	10.7
7	3.186	15.5	3.24	-5.4
8	6.120	10.2	5.93	3.2

[a] assay using PL-SA-sensors.

[b] independently measured by clinical laboratory of hospitals.

[c] the difference of the assay and clinical results divided by the clinical results.
